# Comparison of ultrasound-derived fat fraction and hepatorenal index for quantitative detection of hepatic steatosis: a multicenter MRI-PDFF-referenced study

**DOI:** 10.3389/fnut.2026.1867062

**Published:** 2026-09-08

**Authors:** Yimin Wu, Jiang Cheng, Daojing Xu, Kun Wang, Ziyang Dou, Nianan He, Junli Wang

**Affiliations:** 1 Department of Ultrasound, Wuhu Hospital, East China Normal University (The Second People’s Hospital, Wuhu), Wuhu, China; 2 Department of Ultrasound, Zhongda Hospital, School of Medicine, Southeast University, Nanjing, China; 3 Department of Ultrasound, Bengbu Third People’s Hospital Affiliated to Bengbu Medical College, Bengbu, China; 4 Department of Ultrasound, The First Affiliated Hospital of USTC, Division of Life Sciences and Medicine, University of Science and Technology of China, Hefei, Anhui, China

**Keywords:** hepatic steatosis, hepatorenal index, metabolic dysfunction-associated steatotic liver disease, MRI proton density fat fraction, ultrasound-derived fat fraction

## Abstract

**Background:**

MRI-PDFF enables accurate non-invasive quantification of liver fat, but its routine use is limited by cost, availability, and workflow constraints. Ultrasound-derived fat fraction (UDFF) may offer a more accessible quantitative alternative, yet its added value over the conventional hepatorenal index (HRI) remains uncertain when both are judged against the same MRI-PDFF reference standard.

**Methods:**

This secondary cross-sectional analysis included 137 participants from a prospectively collected three-center cohort with MRI-PDFF, UDFF, and evaluable HRI measurements. Hepatic steatosis was defined as MRI-PDFF ≥5.0%. Diagnostic performance was compared using ROC analysis and paired DeLong testing, with center-stratified and alternative MRI-PDFF threshold analyses. Measurement reproducibility was evaluated for both UDFF and HRI.

**Results:**

MRI-PDFF-defined hepatic steatosis was present in 85 of 137 participants. UDFF showed higher diagnostic discrimination than HRI (AUC, 0.945 [95% CI, 0.907–0.983] vs. 0.779 [95% CI, 0.700–0.859]; paired DeLong *p* = 0.0002), with consistently high center-specific AUCs (0.910–0.960) and similar results across MRI-PDFF thresholds of 5.2, 5.5, and 6.0%. UDFF demonstrated excellent within-examination repeatability and good interobserver reproducibility (ICC, 0.983 and 0.884, respectively); HRI also showed good intraobserver and interobserver reproducibility (ICC, 0.889 and 0.844, respectively).

**Conclusion:**

In this three-center cohort, UDFF provided stronger and more consistent MRI-PDFF-referenced discrimination for hepatic steatosis than HRI, while both methods showed good measurement reproducibility. UDFF may therefore provide a more robust quantitative ultrasound approach for hepatic steatosis assessment.

## 1 Introduction

Metabolic dysfunction-associated steatotic liver disease (MASLD) is a major and growing cause of chronic liver disease. Population-level evidence indicates that steatotic liver disease affects approximately 30% of adults worldwide, with a substantial and increasing burden in China (1, 2). Hepatic steatosis is the defining imaging feature of MASLD, making accurate liver-fat quantification important for characterizing disease burden and evaluating change. Liver biopsy remains the histologic reference but is invasive and affected by sampling and interpretation limitations (3). MRI proton density fat fraction (MRI-PDFF) provides continuous and reproducible liver-fat quantification with established linearity and validation against MR spectroscopy and histologic steatosis (4–6). MRI-PDFF is therefore widely used as a non-invasive reference standard, although cost, scanner availability, and workflow requirements limit routine use.

Ultrasound is more accessible, but available methods differ in measurement principle and technical robustness. Conventional B-mode imaging is qualitative and has limited sensitivity for mild steatosis (7, 8). The hepatorenal index (HRI) quantifies the echogenicity contrast between liver and renal cortex, but its performance depends on acquisition settings, ROI placement, beam path, and the renal cortical reference (7–9). Ultrasound-derived fat fraction (UDFF) combines attenuation and backscatter information to provide a quantitative estimate of liver fat (10–13) and may therefore offer greater robustness than HRI.

Direct comparative evidence supports this possibility. Wang et al. (14) reported that UDFF outperformed controlled attenuation parameter and the hepatic/renal ratio across MRI-PDFF-defined steatosis grades in a single-center cohort. However, the consistency of the UDFF-HRI difference across centers, plausible MRI-PDFF definitions, and repeated measurements remains incompletely characterized. A same-participant multicenter comparison that addresses these sources of uncertainty can therefore clarify the incremental value of UDFF over an accessible B-mode index.

Accordingly, we compared UDFF and HRI for detecting MRI-PDFF-defined hepatic steatosis in adults with suspected MASLD from three centers. We also examined whether the comparative findings were consistent across centers and MRI-PDFF definitions, explored relevant subgroups, and evaluated the measurement reproducibility of both methods.

## 2 Methods

### 2.1 Study design and participants

This secondary cross-sectional analysis used prospectively collected data from adults who were consecutively enrolled at three centers between January and February 2024: Center 1 (Wuhu Hospital, East China Normal University); Center 2 (Bengbu Third People’s Hospital Affiliated to Bengbu Medical College); and Center 3 (The First Affiliated Hospital of USTC). The study was reported in accordance with the STROBE statement. The Institutional Review Board of Wuhu Hospital, East China Normal University approved the study (KY-2024-102). All participants provided written informed consent to participate and for research use of their data. The study followed the Declaration of Helsinki. Eligible participants were adults aged 18 years or older with suspected MASLD, defined as hepatic steatosis on conventional ultrasonography plus at least one cardiometabolic risk factor (overweight or obesity, dysglycemia or type 2 diabetes, hypertriglyceridemia, low high-density lipoprotein cholesterol, or elevated blood pressure) according to the multisociety consensus criteria (15). Exclusion criteria were chronic liver disease of another etiology, alcohol consumption greater than 140 g/week for women or 210 g/week for men, or a contraindication to MRI. MRI-PDFF was performed on the same day as the ultrasound examination or within 1 week. Eligibility for the present secondary analysis additionally required archived liver-kidney B-mode images suitable for offline HRI measurement. No formal *a priori* sample-size calculation was performed; all participants in the parent cohort who met the analysis eligibility criteria were included.

### 2.2 Ultrasound examination, HRI analysis, and reproducibility

**Figure 1:**
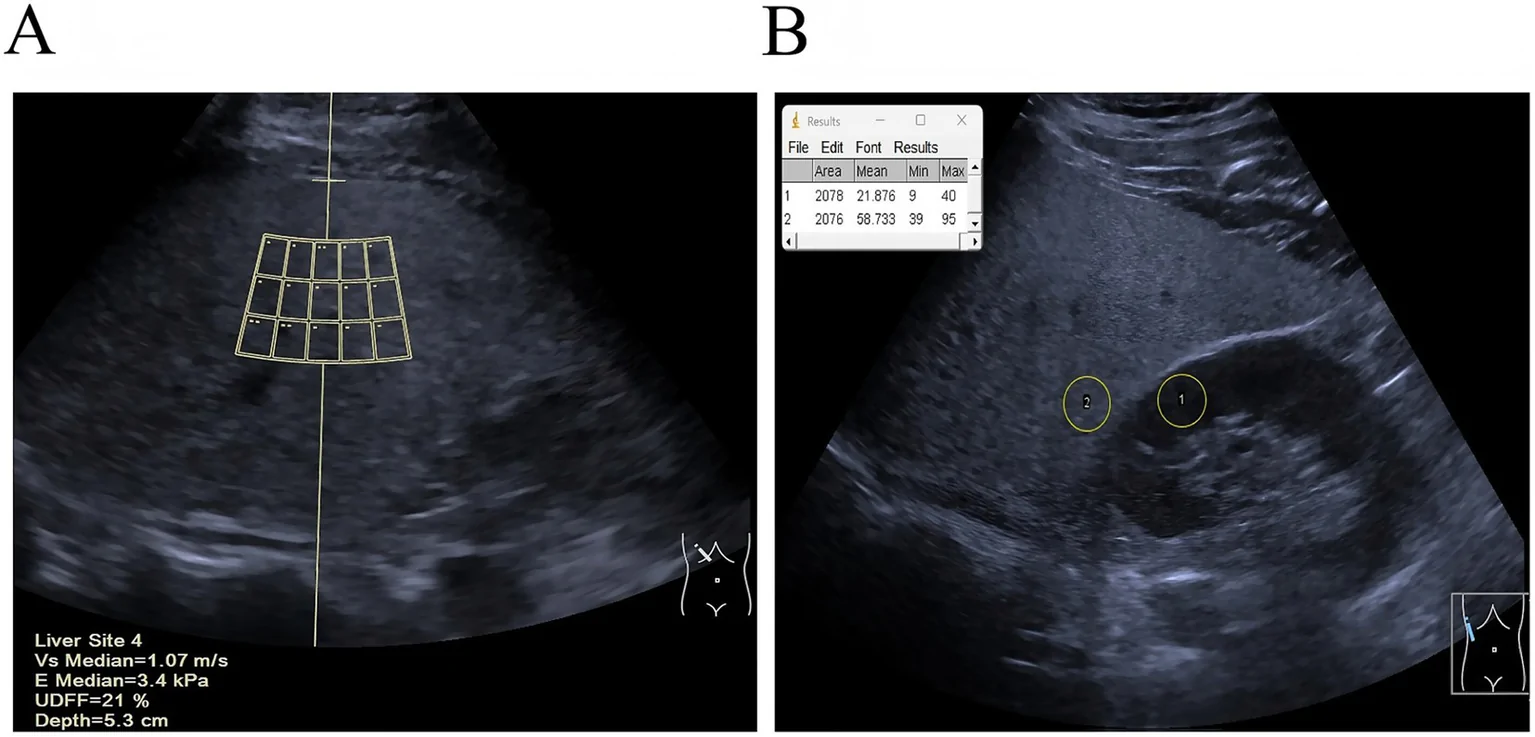
Ultrasound assessment of hepatic steatosis. **(A)** Prospective UDFF acquisition using the dedicated DAX transducer on the ACUSON Sequoia system. **(B)** Offline ImageJ-based HRI measurement from an archived liver-kidney B-mode image acquired using the 5C1 transducer. Equal-sized circular ROIs were placed in the liver parenchyma and right renal cortex at comparable acoustic depths. HRI, hepatorenal index; ROI, region of interest; UDFF, ultrasound-derived fat fraction.

After fasting for at least 4 h, participants were examined in the supine position with the right arm elevated. UDFF was acquired from the right hepatic lobe using the dedicated DAX transducer on the ACUSON Sequoia system (Siemens Healthineers, Erlangen, Germany). The reference line was aligned with the liver capsule, and the ROI was placed in homogeneous hepatic parenchyma, avoiding vessels, bile ducts, rib shadows, and focal artifacts. Five acquisitions were obtained during neutral suspended respiration, and their median was used for diagnostic analyses (Figure 1A). Routine two-dimensional B-mode images of the liver, including a same-frame view of the liver and right kidney, were acquired using the 5C1 curvilinear transducer with a consistent abdominal B-mode preset. For the archived images used for HRI analysis, receiver gain ranged from 0 to 10 dB, and the dynamic range was fixed at 60 dB. Imaging depth was adjusted according to body habitus but did not exceed 15 cm, allowing the liver parenchyma and right kidney to be displayed in the same image. Archived images were analyzed offline using ImageJ (National Institutes of Health, Bethesda, MD, United States). For each examination, three pairs of equal-sized circular ROIs were placed in homogeneous segment VI liver parenchyma and the mid-cortex of the right kidney at comparable acoustic depths within the same image. Large vessels, focal hepatic lesions, renal medulla, renal sinus, collecting system, and imaging artifacts were avoided. For each ROI pair, HRI was calculated as the ratio of the mean hepatic grayscale value to the mean renal cortical grayscale value; the mean of the three pairwise ratios was used for analysis (Figure 1B). At Center 1, YW, with 12 years of ultrasound experience, and DX, with 14 years of ultrasound experience, performed the reproducibility assessments. UDFF within-examination consistency was evaluated in all 41 Center 1 participants using the five acquisitions obtained by YW. Interobserver reproducibility was assessed using participant-level UDFF values independently acquired by the two sonographers. HRI intraobserver and interobserver reproducibility were evaluated in a stratified sample of 42 of 137 evaluable examinations (30.7%). For the intraobserver assessment, YW repeated ROI placement and HRI measurement on the same archived images; for the interobserver assessment, DX independently analyzed the same images. The sonographers performing prospective UDFF acquisitions and the readers performing offline HRI analyses were blinded to MRI-PDFF results and clinical and laboratory data.

### 2.3 MRI-PDFF examination

MRI-PDFF was the non-invasive reference standard because it provides quantitative liver-fat measurements with established linearity, precision, and validation against histology and MR spectroscopy (4, 5). Hepatic steatosis was defined primarily as MRI-PDFF of 5.0% or greater, consistent with the conventional boundary for abnormal liver fat; alternative definitions of 5.2, 5.5, and 6.0% were examined in sensitivity analyses (7, 16). MRI was performed on 3.0-T systems from GE Healthcare (Waukesha, WI) or Siemens Healthineers (Erlangen, Germany) using six-echo chemical shift-encoded gradient-echo imaging with T2* correction and a multipeak fat model. Siemens parameters were repetition time/echo time, 9.3/1.3 ms; matrix, 256 × 192; section thickness, 3 mm; and flip angle, 4 degrees. GE parameters were repetition time, 6.392 ms; first echo time, 1.1 ms with 1.0-ms interecho spacing; matrix, 160 × 144; section thickness, 10 mm; and flip angle, 3 degrees. At each center, one radiologist with at least 3 years of experience in MRI interpretation, blinded to UDFF and laboratory results, placed one largest-fit circular ROI in each of segments V, VI, and VIII on the PDFF map, avoiding vessels, bile ducts, focal lesions, and the liver edge (13, 17, 18). The participant-level MRI-PDFF value used in all analyses was the median of the three segmental measurements.

### 2.4 Statistical analysis

Statistical analyses were performed using Python 3.11.4. Continuous variables are presented as mean ± SD or median (IQR), and categorical variables as number (percentage). Center-level differences in MRI-PDFF and steatosis prevalence were evaluated using the Kruskal-Wallis and Pearson chi-square tests, respectively. Associations with MRI-PDFF were assessed using Spearman correlation, and agreement between UDFF and MRI-PDFF using Bland–Altman analysis. Diagnostic performance was evaluated by ROC analysis; AUCs with 95% CIs were compared between UDFF and HRI using paired DeLong tests. The Youden threshold maximized sensitivity + specificity − 1. Rule-out and rule-in thresholds were the observed cutpoints meeting sensitivity ≥90% and specificity ≥90%, respectively. Wilson 95% CIs were used for sensitivity, specificity, predictive values, and accuracy, and likelihood-ratio CIs were calculated on the log scale. Sensitivity analyses were examined across centers, alternative MRI-PDFF thresholds, and strata of body habitus, lipid status, and skin-to-liver capsule distance, with Chinese adult thresholds used for BMI and waist circumference (19). Measurement reproducibility was assessed using absolute-agreement ICCs. Within-examination UDFF consistency was summarized using a one-way random-effects average-measure ICC for the five-acquisition protocol; the median of the five acquisitions remained the diagnostic value. Interobserver analyses used two-way random-effects single-measure models, and HRI intraobserver analysis used a two-way mixed-effects single-measure model. Bootstrap 95% CIs for correlations and ICCs were based on 2,000 resamples. All tests were two-sided, with *p* < 0.05 indicating statistical significance.

## 3 Results

### 3.1 Participant characteristics

**Figure 2:**
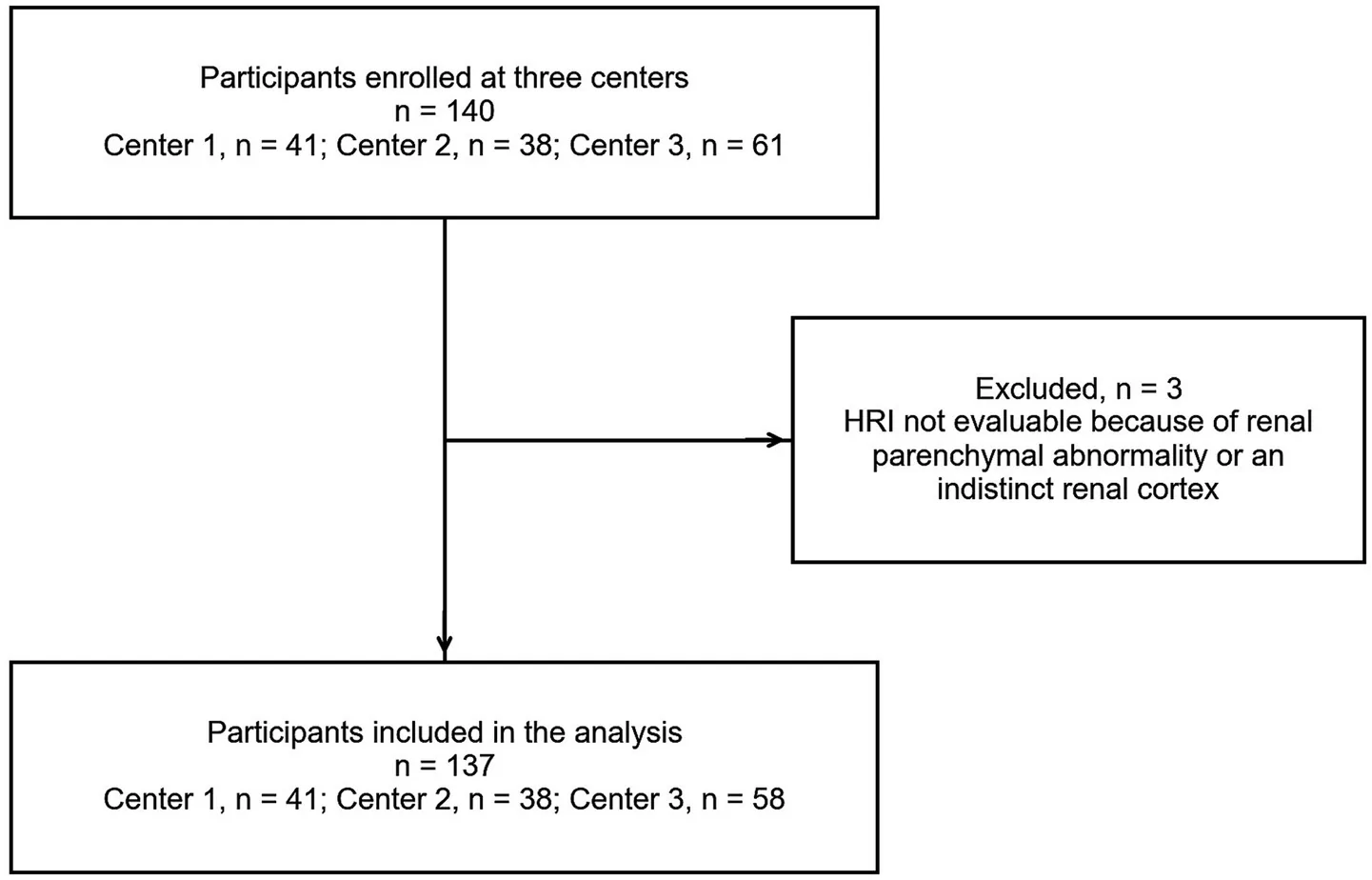
Participant flow for the secondary analysis. During the prospective data-collection period, 140 participants were consecutively enrolled at three centers: 41 at Center 1, 38 at Center 2, and 61 at Center 3. All participants completed MRI-PDFF and UDFF examinations. Three participants from Center 3 were excluded from the comparative UDFF–HRI analysis because HRI could not be reliably evaluated owing to renal parenchymal abnormality or an indistinct renal cortex on B-mode images. The final analysis included 137 participants with MRI-PDFF, UDFF, and evaluable HRI measurements. HRI, hepatorenal index; MRI-PDFF, magnetic resonance imaging proton density fat fraction; UDFF, ultrasound-derived fat fraction.

The prospective parent cohort included 140 consecutively enrolled participants (Center 1, *n* = 41; Center 2, *n* = 38; Center 3, *n* = 61), all of whom completed MRI-PDFF and UDFF examinations. HRI could not be reliably measured from the archived B-mode images in three Center 3 participants because of renal parenchymal abnormality or an indistinct renal cortex. The comparative analysis therefore included 137 participants with MRI-PDFF, UDFF, and evaluable HRI measurements (Figure 2; Table 1). The cohort included 89 men and 48 women; mean age was 42.1 ± 12.7 years, mean BMI was 26.1 ± 3.4 kg/m^2^, and mean waist circumference was 92.0 ± 10.6 cm. Type 2 diabetes was present in 22 of 137 participants (16.1%); operationally defined hypertension was present in 30 of 136 participants with available blood-pressure measurements (22.1%), and 91 of 137 (66.4%) met the lipid-abnormality criterion. MRI-PDFF-defined hepatic steatosis (MRI-PDFF ≥5.0%) was present in 85 of 137 participants (62.0%). The proportion with MRI-PDFF-defined steatosis varied by center: Center 1, 14 of 41 participants (34.1%); Center 2, 29 of 38 participants (76.3%); and Center 3, 42 of 58 participants (72.4%; Pearson chi-square *p* < 0.001). Median MRI-PDFF was 8.28% (IQR, 3.90–13.43%) overall and differed across centers (Center 1, 4.39%; Center 2, 10.62%; Center 3, 8.95%; Kruskal-Wallis *p* < 0.001).

**Table 1:** Baseline characteristics of the analysis cohort by center.

Characteristic	Overall	Center 1	Center 2	Center 3
Participants, *n*	137	41	38	58
Age, years	42.1 ± 12.7	38.6 ± 13.7	47.3 ± 12.6	41.1 ± 10.9
BMI, kg/m^2^	26.1 ± 3.4	24.6 ± 3.3	27.7 ± 3.8	26.2 ± 2.6
Waist circumference, cm	92.0 ± 10.6	85.1 ± 10.9	95.6 ± 11.8	94.4 ± 6.8
Triglycerides, mmol/L	1.54 [1.05, 2.42]	1.05 [0.80, 1.61]	1.96 [1.31, 2.94]	1.69 [1.29, 2.49]
Total cholesterol, mmol/L	4.9 ± 0.9	5.0 ± 1.0	5.0 ± 0.9	4.8 ± 0.8
HDL-C, mmol/L	1.2 ± 0.3	1.2 ± 0.3	1.2 ± 0.3	1.0 ± 0.2
LDL-C, mmol/L	3.0 ± 0.7	2.8 ± 0.7	3.1 ± 0.7	3.0 ± 0.7
Fasting plasma glucose, mmol/L	5.17 [4.86, 5.70]	4.98 [4.69, 5.68]	5.14 [4.82, 5.84]	5.25 [4.92, 5.61]
GHb, %	6.5 ± 0.7	7.1 ± 0.8	6.1 ± 0.6	6.4 ± 0.5
ALT, U/L	29.00 [16.00, 48.00]	16.00 [10.00, 23.00]	39.50 [20.02, 50.90]	32.00 [24.00, 48.00]
AST, U/L	23.00 [19.50, 29.90]	21.00 [18.00, 25.00]	22.90 [19.27, 31.80]	25.50 [21.25, 28.75]
GGT, U/L	30.00 [16.00, 51.00]	16.00 [12.00, 38.00]	37.00 [26.25, 53.00]	30.00 [20.00, 56.50]
Skin-to-liver capsule distance, mm	21.1 ± 3.9	19.5 ± 3.2	22.7 ± 5.0	21.2 ± 3.2
MRI-PDFF, %	8.28 [3.90, 13.43]	4.39 [2.80, 8.60]	10.62 [5.69, 17.94]	8.95 [4.52, 14.20]
UDFF, %	11.00 [7.00, 17.00]	7.00 [4.00, 8.00]	15.00 [10.00, 22.25]	13.00 [9.00, 18.00]
HRI	1.81 [1.51, 2.38]	1.60 [1.29, 2.06]	1.76 [1.46, 2.32]	2.02 [1.65, 2.52]
Male sex	89/137 (65.0%)	18/41 (43.9%)	26/38 (68.4%)	45/58 (77.6%)
Type 2 diabetes	22/137 (16.1%)	5/41 (12.2%)	9/38 (23.7%)	8/58 (13.8%)
Hypertension^a^	30/136 (22.1%)	4/41 (9.8%)	3/38 (7.9%)	23/57 (40.4%)
Lipid-abnormality^b^	91/137 (66.4%)	19/41 (46.3%)	25/38 (65.8%)	47/58 (81.0%)
MRI-PDFF ≥5.0%	85/137 (62.0%)	14/41 (34.1%)	29/38 (76.3%)	42/58 (72.4%)

Values are mean ± SD, median [IQR], or *n*/*N* (%). Center 1, Wuhu Hospital, East China Normal University; Center 2, Bengbu Third People’s Hospital Affiliated to Bengbu Medical College; Center 3, The First Affiliated Hospital of USTC. 𝐻lood-pressure data were unavailable for one participant. Hypertension was defined as systolic blood pressure ≥140 mm Hg or diastolic blood pressure ≥90 mm Hg at enrollment. ᵇLipid abnormality was defined as triglycerides ≥1.70 mmol/L or HDL-C ≤ 1.0 mmol/L in men or ≤1.3 mmol/L in women, according to the multisociety MASLD criteria (15). ALT, alanine aminotransferase; AST, aspartate aminotransferase; BMI, body mass index; GGT, gamma-glutamyl transferase; GHb, glycated hemoglobin; HDL-C, high-density lipoprotein cholesterol; HRI, hepatorenal index; IQR, interquartile range; LDL-C, low-density lipoprotein cholesterol; MRI-PDFF, magnetic resonance imaging proton density fat fraction; SD, standard deviation; UDFF, ultrasound-derived fat fraction.

### 3.2 Diagnostic performance of HRI and UDFF

**Figure 3:**
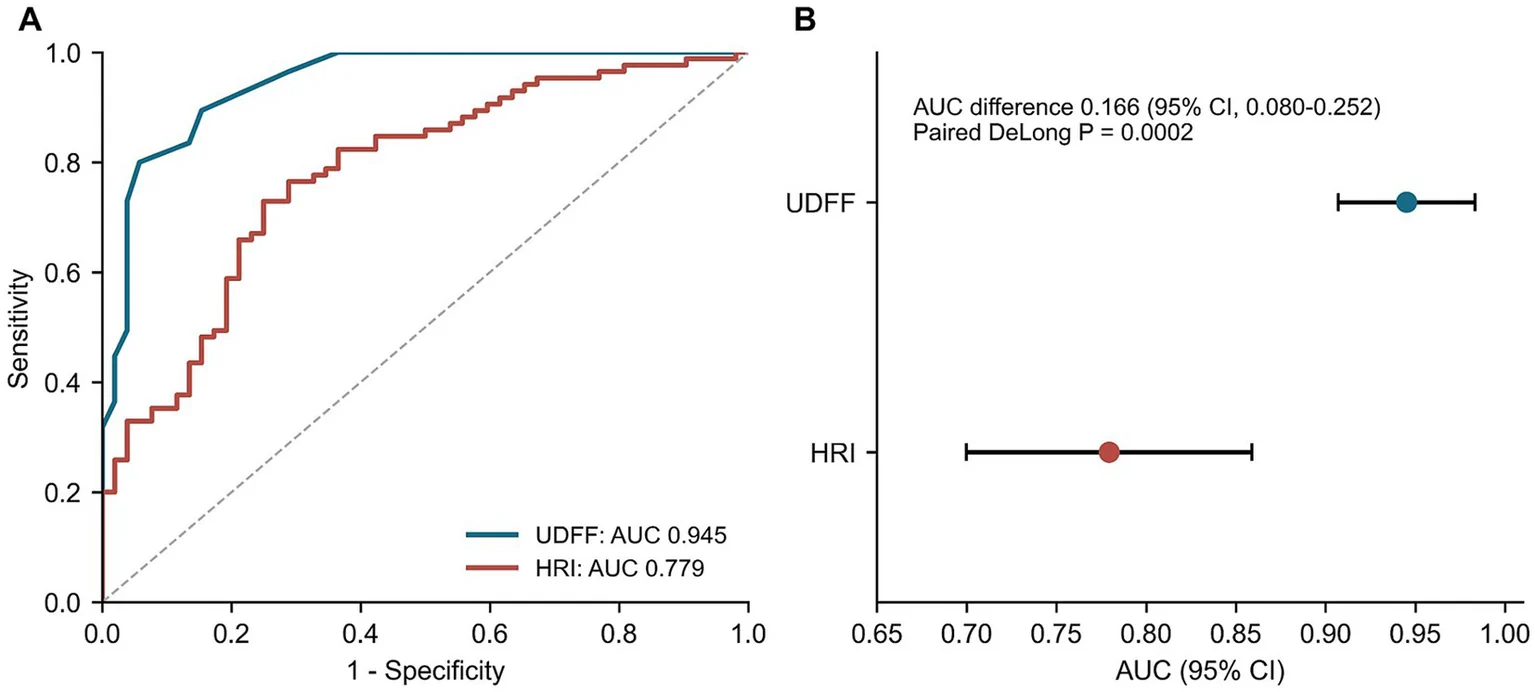
Diagnostic performance of UDFF and HRI for MRI-PDFF-defined hepatic steatosis. **(A)** ROC curves for UDFF and HRI using MRI-PDFF ≥5.0% as the reference definition of hepatic steatosis. The diagonal dashed line indicates the no-discrimination reference. **(B)** AUC estimates with 95% CIs. The paired AUC difference, calculated as UDFF minus HRI, was 0.166 (95% CI, 0.080–0.252; paired DeLong *p* = 0.0002). AUC, area under the receiver operating characteristic curve; CI, confidence interval; HRI, hepatorenal index; MRI-PDFF, magnetic resonance imaging proton density fat fraction; ROC, receiver operating characteristic; UDFF, ultrasound-derived fat fraction.

**Figure 4:**
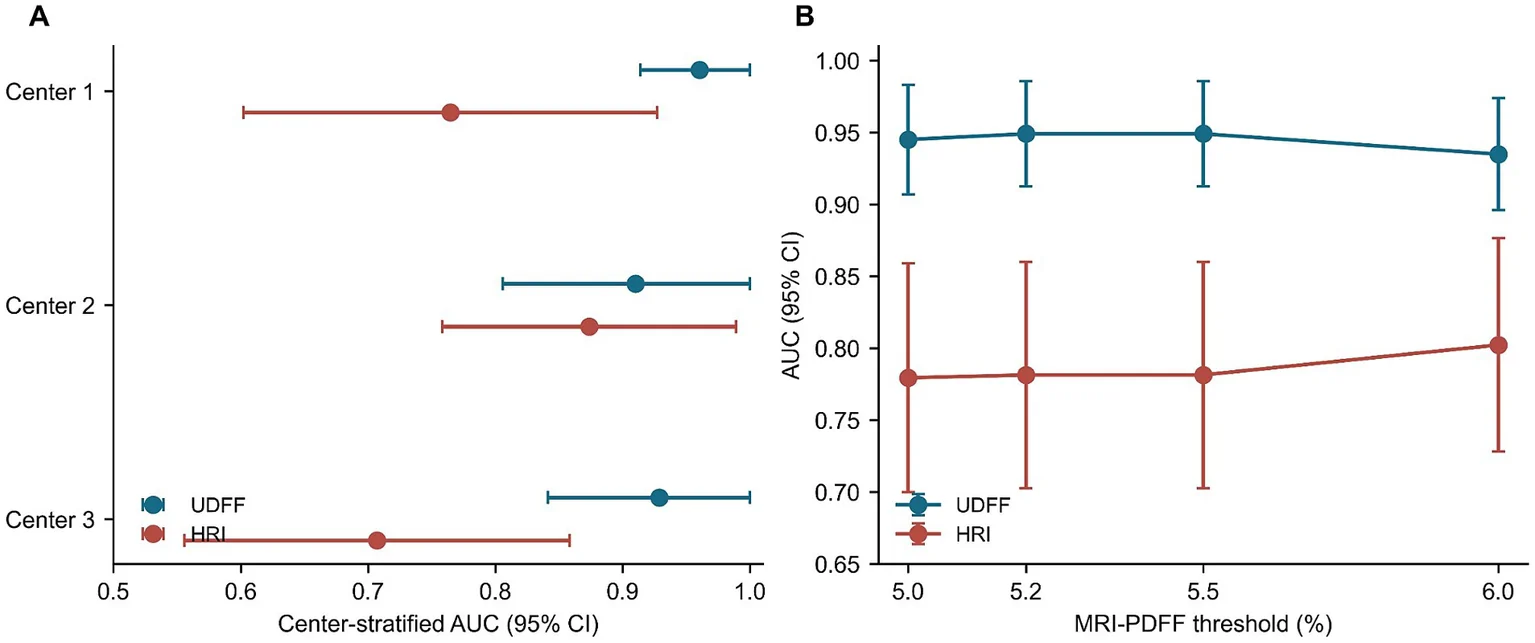
Center-stratified and MRI-PDFF threshold sensitivity analyses. **(A)** Center-specific AUC estimates with 95% CIs for UDFF and HRI. **(B)** AUC estimates with 95% CIs using MRI-PDFF thresholds of 5.0, 5.2, 5.5, and 6.0% to define hepatic steatosis. AUC, area under the receiver operating characteristic curve; CI, confidence interval; HRI, hepatorenal index; MRI-PDFF, magnetic resonance imaging proton density fat fraction; UDFF, ultrasound-derived fat fraction.

Representative UDFF and HRI measurements are shown in Figure 1. Using MRI-PDFF ≥5.0% as the primary reference threshold, UDFF showed higher diagnostic discrimination than HRI for detecting hepatic steatosis (AUC, 0.945 [95% CI, 0.907–0.983] vs. 0.779 [95% CI, 0.700–0.859]; AUC difference, 0.166 [95% CI, 0.080–0.252]; paired DeLong *p* = 0.0002; Figure 3). Full threshold-based diagnostic metrics, including sensitivity, specificity, PPV, NPV, LR+, LR−, and accuracy with 95% CIs, are shown in Table 2. The UDFF Youden and rule-in thresholds coincided at 10.5%, whereas the rule-out threshold was 7.5%. Across MRI-PDFF threshold sensitivity analyses at 5.2, 5.5, and 6.0%, UDFF maintained higher AUCs than HRI, with paired DeLong *p* values ranging from 0.0001 to 0.0016 (Figure 4B; Table 3).

**Table 2:** Threshold-based diagnostic performance of UDFF and HRI for MRI-PDFF-defined hepatic steatosis.

Method and threshold	Sensitivity/Specificity, % (95% CI)	PPV/NPV, % (95% CI)	LR+/LR− (95% CI)	Accuracy, % (95% CI)
UDFF, Youden index (10.5%)	Sens 80.0 (70.3–87.1); Spec 94.2 (84.4–98.0)	PPV 95.8 (88.3–98.6); NPV 74.2 (62.6–83.3)	LR + 13.87 (4.60–41.81); LR- 0.21 (0.14–0.33)	85.4 (78.5–90.3)
UDFF, rule-out (7.5%)	Sens 96.5 (90.1–98.8); Spec 71.2 (57.7–81.7)	PPV 84.5 (76.0–90.4); NPV 92.5 (80.1–97.4)	LR + 3.34 (2.18–5.13); LR- 0.05 (0.02–0.15)	86.9 (80.2–91.5)
UDFF, rule-in (10.5%)	Sens 80.0 (70.3–87.1); Spec 94.2 (84.4–98.0)	PPV 95.8 (88.3–98.6); NPV 74.2 (62.6–83.3)	LR + 13.87 (4.60–41.81); LR- 0.21 (0.14–0.33)	85.4 (78.5–90.3)
HRI, Youden index (1.77)	Sens 72.9 (62.7–81.2); Spec 75.0 (61.8–84.8)	PPV 82.7 (72.6–89.6); NPV 62.9 (50.5–73.8)	LR + 2.92 (1.79–4.75); LR- 0.36 (0.25–0.53)	73.7 (65.8–80.4)
HRI, rule-out (1.44)	Sens 90.6 (82.5–95.2); Spec 40.4 (28.2–53.9)	PPV 71.3 (62.1–79.0); NPV 72.4 (54.3–85.3)	LR + 1.52 (1.20–1.92); LR- 0.23 (0.11–0.49)	71.5 (63.5–78.4)
HRI, rule-in (2.39)	Sens 35.3 (26.0–45.9); Spec 92.3 (81.8–97.0)	PPV 88.2 (73.4–95.3); NPV 46.6 (37.3–56.2)	LR + 4.59 (1.71–12.28); LR- 0.70 (0.59–0.84)	56.9 (48.6–64.9)

Sensitivity, specificity, PPV, NPV, and accuracy are presented as percentages with 95% CIs; LR+ and LR− are presented as ratios with 95% CIs. Hepatic steatosis was defined as MRI-PDFF ≥5.0%. The Youden threshold maximized sensitivity + specificity − 1. Rule-out and rule-in thresholds were defined by sensitivity ≥90% and specificity ≥90%, respectively. Wilson score CIs were used for proportions, and CIs for likelihood ratios were calculated using the log method. CI, confidence interval; HRI, hepatorenal index; LR+, positive likelihood ratio; LR−, negative likelihood ratio; MRI-PDFF, magnetic resonance imaging proton density fat fraction; NPV, negative predictive value; PPV, positive predictive value; Sens, sensitivity; Spec, specificity; UDFF, ultrasound-derived fat fraction.

**Table 3:** MRI-PDFF threshold sensitivity analyses for UDFF and HRI.

MRI-PDFF threshold	Positive/Negative cases	UDFF AUC (95% CI)	HRI AUC (95% CI)	AUC difference (95% CI)	*P* value
5.0%	85/52	0.945 (0.907–0.983)	0.779 (0.700–0.859)	0.166 (0.080–0.252)	0.0002
5.2%	84/53	0.949 (0.913–0.986)	0.781 (0.703–0.860)	0.168 (0.083–0.253)	0.0001
5.5%	84/53	0.949 (0.913–0.986)	0.781 (0.703–0.860)	0.168 (0.083–0.253)	0.0001
6.0%	79/58	0.935 (0.896–0.974)	0.802 (0.728–0.877)	0.133 (0.050–0.215)	0.0016

AUCs and between-method differences are presented with DeLong 95% CIs. AUC differences were calculated as UDFF minus HRI. *p*-values were obtained using paired DeLong tests. AUC, area under the receiver operating characteristic curve; CI, confidence interval; HRI, hepatorenal index; MRI-PDFF, magnetic resonance imaging proton density fat fraction; UDFF, ultrasound-derived fat fraction.

### 3.3 Center- and subgroup-stratified analyses

Despite differences in MRI-PDFF distribution and steatosis prevalence across centers, UDFF showed consistently high AUCs: Center 1, 0.960 (95% CI, 0.914–1.000); Center 2, 0.910 (95% CI, 0.806–1.000); and Center 3, 0.929 (95% CI, 0.841–1.000). HRI AUCs varied more across centers: Center 1, 0.765 (95% CI, 0.602–0.927); Center 2, 0.874 (95% CI, 0.758–0.989); and Center 3, 0.707 (95% CI, 0.556–0.858) (Figure 4A). Across strata of BMI, waist circumference, lipid status, and skin-to-liver capsule distance, UDFF had a numerically higher AUC than HRI in every subgroup (UDFF range, 0.896–1.000; HRI range, 0.684–0.873; Supplementary Table 1). UDFF AUCs were 1.000 below and 0.896 at or above a BMI of 24 kg/m^2^, 0.985 without and 0.903 with central obesity, 0.935 and 0.961 across lipid-status strata, and 0.935 and 0.939 at skin-to-liver capsule distances of 21 mm or less and greater than 21 mm, respectively.

### 3.4 Measurement reproducibility

In the 41 participants from Center 1, UDFF demonstrated excellent within-examination repeatability (ICC, 0.983; bootstrap 95% CI, 0.965–0.990) and good interobserver reproducibility (ICC, 0.884; bootstrap 95% CI, 0.758–0.938). In the 42-participant HRI subset, intraobserver reproducibility was good (ICC, 0.889; bootstrap 95% CI, 0.809–0.938), as was interobserver reproducibility (ICC, 0.844; bootstrap 95% CI, 0.708–0.915) (Table 4).

**Table 4:** Repeatability and reproducibility of UDFF and HRI.

Measure	Analysis	n	ICC (95% CI)	Bias	95% LoA
UDFF	Within-examination repeatability	41	0.983 (0.965–0.990)	Not applicable	Not applicable
UDFF	Interobserver reproducibility	41	0.884 (0.758–0.938)	0.76	−3.94 to 5.45
HRI	Intraobserver reproducibility	42	0.889 (0.809–0.938)	−0.016	−0.634 to 0.603
HRI	Interobserver reproducibility	42	0.844 (0.708–0.915)	0.025	−0.746 to 0.795

ICCs are absolute-agreement estimates with bootstrap 95% CIs. Bias and 95% LoA are expressed in percentage points for UDFF and in HRI units for HRI. CI, confidence interval; HRI, hepatorenal index; ICC, intraclass correlation coefficient; LoA, limits of agreement; UDFF, ultrasound-derived fat fraction.

### 3.5 Correlation and agreement with MRI-PDFF

**Figure 5:**
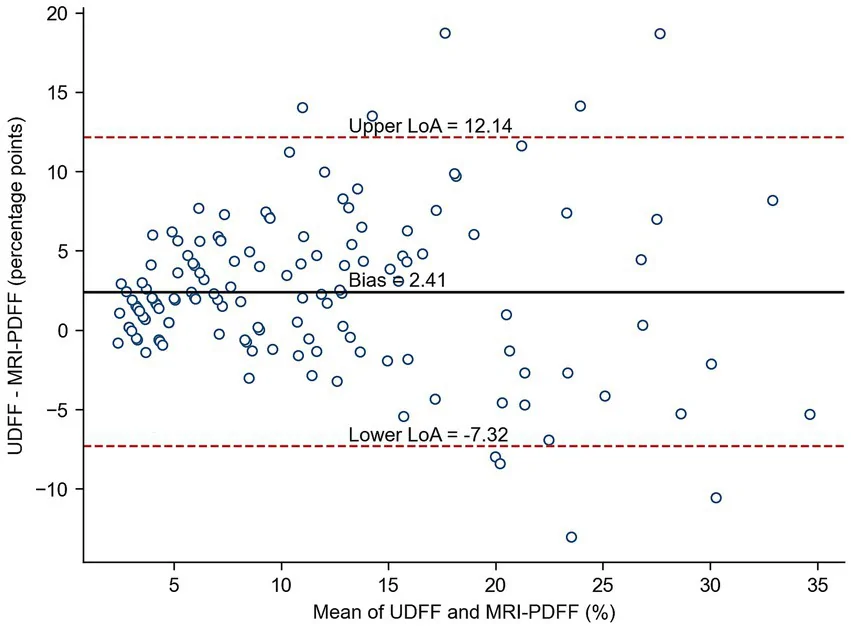
Bland–Altman analysis of agreement between UDFF and MRI-PDFF in 137 participants. Each point represents one participant. The solid horizontal line indicates the mean UDFF-minus-MRI-PDFF bias of 2.41 percentage points, and the dashed horizontal lines indicate the 95% LoA, ranging from −7.32 to 12.14 percentage points. LoA, limits of agreement; MRI-PDFF, magnetic resonance imaging proton density fat fraction; UDFF, ultrasound-derived fat fraction.

Both UDFF and HRI were correlated with MRI-PDFF. UDFF showed a strong correlation with MRI-PDFF (Spearman rho, 0.845; bootstrap 95% CI, 0.790–0.883), whereas HRI showed a moderate correlation (Spearman rho, 0.509; bootstrap 95% CI, 0.376–0.627). These relationships are shown in Supplementary Figure 1. Bland–Altman analysis showed that UDFF slightly overestimated MRI-PDFF, with a mean bias of 2.41 percentage points and 95% limits of agreement from −7.32 to 12.14 percentage points (Figure 5).

## 4 Discussion

In this three-center cohort of adults with suspected MASLD, UDFF showed greater discrimination than HRI for MRI-PDFF-defined hepatic steatosis. This pattern was observed across centers and at all evaluated MRI-PDFF thresholds from 5.0 to 6.0%, and UDFF had a numerically higher AUC than HRI in every exploratory subgroup defined by BMI, central obesity, lipid status, or skin-to-liver capsule distance. UDFF demonstrated excellent within-examination repeatability and good interobserver reproducibility, while offline HRI measurements showed good intraobserver and interobserver reproducibility. Taken together, the primary comparison and supporting analyses consistently favored UDFF over HRI.

The magnitude of UDFF performance was consistent with, and in some settings higher than, previous MRI-PDFF-referenced studies. Dillman et al. (12) reported an AUC of 0.90 for detecting MRI-PDFF of at least 5.5%, whereas De Robertis et al. (20) reported an AUC of 0.75 using a 5% reference threshold. In a single-center direct comparison, Wang et al. (14) found an AUC of 0.99 for UDFF and 0.87 for the hepatic/renal ratio at MRI-PDFF of at least 6%. The present AUCs of 0.945 for UDFF and 0.779 for HRI therefore fall within the published performance range while adding a paired comparison across three centers. The less stable center-specific HRI estimates are also consistent with prospective HRI studies showing that the method can detect the presence of steatosis but is less reliable for separating higher grades (9, 21).

Our 10.5% Youden threshold was higher than the 5% threshold reported by Dillman et al. (12), the 6% threshold reported by Wang et al. (14), and the 8% threshold validated by Xue et al. (22). These values are not directly interchangeable: the corresponding MRI-PDFF definitions were 5.5, 6.0, and 5.75%, respectively, and the studies differed in cohort spectrum and threshold objective. Xue et al. (22) distinguished a Youden threshold of 8% from rule-out and rule-in thresholds of 6 and 10%; in our cohort, the rule-out threshold was 7.5%, while the Youden and rule-in thresholds coincided at 10.5%. A recent meta-analysis of nine MRI-PDFF-referenced studies reported pooled sensitivity of 90.4%, pooled specificity of 83.8%, and a summary AUC of 0.93; eight of the nine studies used the same vendor platform (23). This supports high overall diagnostic performance while reinforcing the need to validate decision thresholds across populations and platforms. In the present study, 10.5% was derived only under the prespecified primary MRI-PDFF definition of 5.0%. The alternative MRI-PDFF thresholds were used to test the robustness of the paired AUC comparison, not to generate multiple *post hoc* clinical cutoffs in this sample. Thus, the preserved UDFF-HRI AUC difference supports the comparative conclusion, whereas numerical thresholds remain dependent on the reference definition, cohort, and decision objective.

The HRI results reflect limitations intrinsic to using renal cortex as an internal reference. Although placing liver and renal ROIs at similar depths reduces differential beam attenuation, HRI remains sensitive to ROI placement, intervening attenuation, respiratory acquisition, receiver gain, dynamic range, time-gain compensation, and machine-dependent image processing (7, 8, 21). Gao et al. (24) showed that renal cortical backscatter anisotropy produced position-dependent HRI thresholds and prevented reliable HRI acquisition in 8.2% of examinations. Renal parenchymal alteration can also change cortical echogenicity and destabilize the denominator independently of liver fat (7, 21). Thus, good reproducibility of standardized ImageJ ROI measurements does not eliminate variability introduced during B-mode acquisition or by the renal reference itself.

The reproducibility findings place this comparison in the context of prior UDFF studies. Xue et al. (22) reported intraobserver and interobserver ICCs of 0.985 and 0.971, and Song et al. (25) reported corresponding ICCs of 0.96 and 0.94. Our within-examination ICC of 0.983 was similar, whereas the interobserver ICC of 0.884 was lower but remained good. Because the average-measure ICC summarized the five-acquisition examination protocol whereas the diagnostic value was the median of five acquisitions, this estimate should be interpreted as protocol-level consistency rather than reliability of a single acquisition. The lower interobserver ICC may reflect the use of independently performed examinations rather than repeated analysis of the same acquisition. Reproducibility is also platform-specific: Jeon et al. (26) found a mean difference of 1.3 percentage points and limits of agreement from −8.0 to 10.6 between two ultrasound fat-fraction platforms, indicating that values from different vendors should not be treated as interchangeable.

The Bland–Altman findings further separate diagnostic discrimination from numerical interchangeability. The mean UDFF-minus-MRI-PDFF bias of 2.41 percentage points was smaller than the 4.0-point bias reported by Dillman et al. (12) and similar in direction to the 1.5-point bias reported by Kubale et al. (13) and the 1.33-point bias reported by Xue et al. (22). However, our limits of agreement (−7.32 to 12.14) were comparable to those in Xue et al. (22) (−9.32 to 11.97) and Kubale et al. (13) (−11.0 to 14.0), showing that modest average bias can coexist with clinically relevant individual differences. Such differences can change classification near the 5% boundary; UDFF and MRI-PDFF should therefore not be used interchangeably for an individual patient. UDFF remained more discriminative than HRI across BMI, waist-circumference, lipid, and skin-to-liver distance strata. Prior studies have reported inconsistent associations of body habitus and skin-to-liver capsule distance with agreement between UDFF and MRI-PDFF across cohorts and ultrasound platforms (12, 22, 26). Because attenuation measurements decrease with increasing ROI depth, acquisition protocols should continue to standardize liver-capsule-relative ROI placement (27).

This study has limitations. The sample size was determined by eligible participants in the parent cohort, which comprised Chinese adults with suspected MASLD, and the three centers did not constitute an independent validation cohort; confidence intervals therefore define the precision of the estimates, and the thresholds require confirmation in larger independent cohorts, other populations, and other health-care settings. The subgroup analyses were exploratory and were not designed to test effect modification. Skin-to-liver capsule distance was a proxy for the prehepatic acoustic path rather than a direct measurement of ROI depth. UDFF reproducibility was assessed at the principal center on a single-vendor platform, supporting protocol-specific consistency but not cross-platform reproducibility. MRI-PDFF rather than histology served as the reference standard; however, it is a validated quantitative measure of liver fat, and the right-lobe sampling strategy was anatomically aligned with the ultrasound examination (4–6, 18).

In conclusion, UDFF showed stronger and more consistent MRI-PDFF-referenced discrimination than HRI in this three-center cohort, with favorable measurement reproducibility. These findings support UDFF as a more robust quantitative ultrasound approach for detecting hepatic steatosis, while numerical cutoffs and cross-platform use should be interpreted within the acquisition protocol and population in which they were derived.

## Data Availability

The raw data supporting the conclusions of this article will be made available by the authors, without undue reservation.
