# Supplementary material for: Comparison of ultrasound-derived fat fraction and hepatorenal index for quantitative detection of hepatic steatosis: a multicenter MRI-PDFF-referenced study
Source: Front Nutr. 2026 Sep 8;13:1867062. doi: 10.3389/fnut.2026.1867062 (PMC13593891; doi:10.3389/fnut.2026.1867062)
Supplement: Supplementary file 1 [file Supplementary_File_1.docx]

**Supplementary Material**

Comparison of Ultrasound-Derived Fat Fraction and Hepatorenal Index for Quantitative Detection of Hepatic Steatosis: A Multicenter MRI-PDFF-Referenced Study

**Supplementary Table 1. Subgroup analyses of the diagnostic performance of UDFF and HRI stratified by body mass index, central obesity, lipid status, and skin-to-liver capsule distance.**

| **Subgroup** | **Participants, n (steatosis/nonsteatosis)** | **UDFF AUC (95% CI)** | **HRI AUC (95% CI)** | **AUC difference (95% CI)** | **P value** | **UDFF bias (95% LoA)** |
| --- | --- | --- | --- | --- | --- | --- |
| BMI <24 kg/m² | 35, 12/23 | 1.000 (1.000-1.000) | 0.873 (0.743-1.000) | 0.127 (-0.003-0.257) | 0.0558 | 1.57 (-4.48 to 7.61) |
| BMI ≥24 kg/m² | 102, 73/29 | 0.896 (0.828-0.964) | 0.746 (0.641-0.851) | 0.150 (0.027-0.274) | 0.0173 | 2.70 (-7.98 to 13.37) |
| No central obesity | 45, 17/28 | 0.985 (0.960-1.000) | 0.809 (0.683-0.934) | 0.176 (0.046-0.307) | 0.0081 | 0.63 (-5.30 to 6.57) |
| Central obesity | 92, 68/24 | 0.903 (0.831-0.975) | 0.774 (0.668-0.880) | 0.129 (0.009-0.249) | 0.0352 | 3.28 (-7.47 to 14.03) |
| No lipid abnormality | 46, 19/27 | 0.935 (0.866-1.000) | 0.684 (0.518-0.850) | 0.250 (0.079-0.422) | 0.0041 | 2.75 (-5.31 to 10.80) |
| Lipid abnormality | 91, 66/25 | 0.961 (0.927-0.994) | 0.819 (0.718-0.920) | 0.142 (0.036-0.248) | 0.0086 | 2.24 (-8.26 to 12.74) |
| Skin-to-liver capsule distance ≤21 mm | 73, 34/39 | 0.935 (0.880-0.990) | 0.776 (0.668-0.884) | 0.159 (0.048-0.270) | 0.0052 | 2.19 (-5.12 to 9.50) |
| Skin-to-liver capsule distance >21 mm | 63, 51/12 | 0.939 (0.867-1.000) | 0.835 (0.700-0.970) | 0.104 (-0.048-0.256) | 0.1805 | 2.72 (-9.31 to 14.74) |

AUCs and paired AUC differences (UDFF minus HRI) are reported with DeLong 95% CIs; P values were obtained using paired DeLong tests. Subgroups were defined as follows: BMI <24 versus ≥24 kg/m²; central obesity, waist circumference ≥90 cm in men or ≥85 cm in women; lipid abnormality, triglycerides ≥1.70 mmol/L or HDL-C ≤1.0 mmol/L in men or ≤1.3 mmol/L in women; and skin-to-liver capsule distance ≤21 versus >21 mm. Bland–Altman bias and 95% LoA represent UDFF minus MRI-PDFF and are expressed in percentage points. These analyses were exploratory, with no adjustment for multiple comparisons. AUC = area under the receiver operating characteristic curve; BMI = body mass index; CI = confidence interval; HDL-C = high-density lipoprotein cholesterol; HRI = hepatorenal index; LoA = limits of agreement; MRI-PDFF = MRI proton density fat fraction; UDFF = ultrasound-derived fat fraction.
**Supplementary Figure 1**

Supplementary Figure 1. Correlations of MRI-PDFF with UDFF and HRI. Scatterplots show the relationships of MRI-PDFF with (A) UDFF and (B) HRI in 137 participants. Spearman correlation coefficients are shown in each panel.
